# VEGFC/VEGFR3 axis mediates TGFβ1-induced epithelial-to-mesenchymal transition in non-small cell lung cancer cells

**DOI:** 10.1371/journal.pone.0200452

**Published:** 2018-07-11

**Authors:** Lincan Duan, Lianhua Ye, Li Zhuang, Xiaolan Zou, Shan Liu, Yong Zhang, Lijuan Zhang, Congguo Jin, Yunchao Huang

**Affiliations:** 1 Department of Thoracic Surgery, The Third Affiliated Hospital of Kunming Medical University, Kunming, China; 2 Department of Palliative Medicine, The Third Affiliated Hospital of Kunming Medical University, Kunming, China; 3 Cancer Center of Integrative Medicine, The Third Affiliated Hospital of Kunming Medical University, Kunming, China; 4 Department of Pathology, The Third Affiliated Hospital of Kunming Medical University, Kunming, China; 5 Cancer Institute, The Third Affiliated Hospital of Kunming Medical University, Kunming, China; University of South Alabama Mitchell Cancer Institute, UNITED STATES

## Abstract

In the tumor progression, transforming growth factor β1 (TGFβ1) plays a critical role in tumorigenesis as well as metastasis. It is known that high plasma level of TGFβ1 in patients with advanced non-small cell lung cancer (NSCLC) is correlated with poor prognostics. In addition, the generation of cancer stem-like cells is associated with metastasis, drug resistance, and tumor recurrence, which also lead to poor outcomes in NSCLC patients. However, it remains unclear how TGFβ1 promotes NSCLC cells to acquire stem-like properties and accelerate tumor metastasis. In our study, we found that short term TGFβ1 treatment resulted in a significant epithelial-mesenchymal transition (EMT) morphological change in TGFβ1–sensitive NSCLC cells but not in insensitive cells. Western blotting confirmed increased Vimentin and reduced E-Cadherin protein expression after TGFβ1 treatment in A549, NCI-H1993, and NCI-H358 cells. TGFβ1 incubation dramatically decreased *in vitro* cell proliferation and increased cell invasion in TGFβ1–sensitive NSCLC cells but not in NCI-H1975, NCI-H1650, and HCC827 cells. Moreover, TGFβ1 was able to enhance the mRNA expression of Oct4, Nanog and Sox2 and drastically increased anchorage-independent colony formation in TGFβ1–sensitive NSCLC cells, suggesting the acquisition of cancer stem-like properties. Interestingly, we found that vascular endothelial growth factor receptor 3 (VEGFR3) mRNA expression was significantly elevated in TGFβ1–sensitive NSCLC cells compared to insensitive cells. And TGFβ1 was capable of inducing VEGF-C gene expression. Pharmacological blocking TGFβ type I receptor kinase (ALK5) significantly inhibited TGFβ1-induced VEGF-C expression. Silencing of ALK5 by siRNA also dramatically reduced TGFβ1-induced VEGF-C expression in TGFβ1–sensitive NSCLC cells. Therefore, TGFβ1 contributes for NSCLC metastasis through promoting EMT, generation of high invasive cancer cells with stem-like properties, and increasing VEGF-C expression. Blocking TGFβ pathway is a potential therapeutic target in human non-small cell lung cancer.

## Introduction

NSCLC is one of the deadliest cancers worldwide with 5-year overall survival rate of around 16% for decades [1, 2]. One major reason is tumor metastasis and/or recurrence, which is a complex process driven by abnormal activation or suppression of many signal transduction pathways. Among them, TGFβ signaling pathway is one of the most frequently dysregulated pathways. TGFβ is a critical tumor suppressor of epithelial cell proliferation and primary tumorigenesis. However, it is also known as a positive contributor of tumor progression and metastasis because many studies demonstrated that TGFβ can induce EMT in certain types of cancer cells [3]. Two major signaling pathways have been identified as mediators of TGFβ–induced EMT. One is that TGFβ induces EMT via Smad protein mediated TGFβ type I receptor kinase (ALK-5) activation, which facilitates cell motility. Another is that TGFβ-induced EMT involves Ras homolog gene family, member A (RhoA) and p38 mitogen-activated protein kinase (MAPK) pathway activation [4]. Furthermore, certain types of cancer cells induced to undergo EMT showed stem cell-like properties, such as self-renewal and tumor formation. For example, breast cancer stem cells expressing high CD44 and low CD24 exhibit EMT features [5]. Therefore, it is well accepted that EMT is involved in the generation of highly invasive cells bearing cancer stem cell-like features. In certain NSCLC cells, we observed similar results of TGFβ1-induced EMT and generation of lung cancer stem-like cells. We aimed to identify the mechanisms through which TGFβ1 activates and sustains pro-metastatic process.

Vascular endothelial growth factor (VEGF) is an important growth factor family involved in the regulation of numerous cellular events related to angiogenesis, vasculogenesis, and lymphangiogenesis [6, 7]. The mammalian VEGF family includes five ligands VEGF-A, -B, -C, -D and placental growth factor, which bind to their receptors VEGFR1, VEGFR2 and VEGFR3, respectively. VEGF-A binding to VEGFR2 is the key signaling pathway mediating angiogenesis through enhancing endothelial cell proliferation, survival, cell migration and vascular permeability [8]. VEGF-B binding to VEGFR1 promotes the survival of endothelial cells, pericytes, and smooth muscle cells [8]. VEGF-C and VEGF-D bind to VEGFR2 and VEGFR3. Several labs have reported that VEGF-C gene expression level is associated with advanced metastasis in colorectal cancer and to play a role in lymphangiogenesis in multiple types of cancer, including colorectal, lung and breast cancer [9, 10]. VEGF-D is also involved in lymphangiogenesis and lymphatic metastasis [11]. In the current paper, we demonstrated that TGFβ1 can induce EMT and promote the acquisition of cancer stem-like properties in a group of TGFβ1-sensitive NSCLC cells with upregulation of VEGFR3 expression.

## Materials and methods

### Cell culture and antibodies

All human NSCLC cell lines (NCI-H1993, A549, NCI-H358, NCI-H1975, NCI-H1650, HCC827) used in this study were purchased from American Type Culture Collection (Manassas, VA, USA). These NSCLC cell lines were maintained in RPMI-1640 (Sigma-Aldrich, Merck KGaA, Darmstadt, Germany) supplemented with 5% fetal bovine serum (FBS) and cultured at 37 °C in a humidified atmosphere containing 5% CO_2_. Antibodies used in western blotting were purchased from the following companies: anti-ERK1/2 (M5670, rabbit, Sigma-Aldrich, Merck KGaA); anti-phospho-ERK1/2 (Thr202/ Tyr204) (9101S, rabbit, Cell Signaling Technology, Danvers, MA, USA); anti-Cadherin (ab15148, rabbit, Abcam, Cambridge, MA, USA); anti-Vimentin (ab92547, rabbit, Abcam); anti-Actin (ab3280, mouse, Abcam). Reagents used in the study were from the following companies: human recombinant TGFβ1 (T7039, Sigma Aldrich, Merck KGaA), human recombinant VEGF-C (SRP3184, Sigma Aldrich, Merck KGaA), and LY2157299 (S2230, Selleckchem, Houston, TX, USA).

### Quantitative real-time PCR

Total RNA of collected human NSCLC cells were isolated with Trizol reagent (Invitrogen, Carlsbad, CA, USA) according to the manufacturer’s instructions. cDNA was generated with oligo-dT primer from 0.5 μg RNA using an iScript Complementary DNA Synthesis Kit (Bio-Rad Laboratories, Inc., Hercules, CA, USA). TaqMan probes (Thermo Fisher Scientific, Inc., Waltham, MA, USA) of E-cadherin (Hs01023895_m1), Vimentin (Hs00418522_m1), Oct-4 (Hs04260367_gH), Nanog (Hs02387400_g1), Sox2 (Hs01053049_s1), VEGFR3 (Hs01047677_m1), and VEGF-C (Hs01099203_m1) genes were utilized for quantitative analysis of mRNA transcript levels. GAPDH gene was used as an internal control (Hs002786624_g1). Real-time PCR reactions were conducted in Applied Biosystems 7300 real-time PCR system (Applied Biosystems; Thermo Fisher Scientific, Inc.) and the thermocycling conditions were 95°C 3min, 40 cycles of 95°C 15sec and 60°C 45sec. The specific gene expression level was calculated using the ΔΔCt method with Software SDS v1.4.1 [12].

### Western blotting

Collected cells were lysed in Protein Extraction Reagent Type 4 (Sigma-Aldrich, Merck KGaA) with PhosStop phosphatase inhibitor and cOmplete protease inhibitor (Roche Diagnostics, Indianapolis, IN, USA). The protein concentration of cell lysates was measured with the Bradford reagent (Bio-Rad Laboratories, Inc.). Fifty microgram of total protein was electrophoresed through 10% SDS-PAGE gels and was transferred to nitrocellulose membranes (Bio-Rad Laboratories, Inc.) using an electrophoretic transfer chamber (Millipore, Temecula, CA, USA). The blots were incubated with primary antibodies overnight at 4°C followed by incubating with relevant HRP-conjugated secondary antibodies (Goat anti-mouse IgG antibody (Abcam ab6789) and goat anti-rabbit IgG antibody (Abcom ab97051)) for 1 hour at room temperature. Actin (sc-4778, Santa Cruz Biotechnology, Dallas, TX, USA) was used as a loading control. The signal was detected using an ECL western blotting detection kit (Promega, Fitchburg, WI, USA).

### Cell viability assay

Cell viability was detected using CellTiter-Glo luminescent assay (Promega) based on quantitation of ATP level inside live cells. NSCLC cells were cultured in a 96-well plate at a density of 2x10^3^ cells per well. After 72 hours, fresh medium containing CellTiter-Glo substrate was added into the wells, and incubated at room temperature for fifteen minutes. Optical density was measured at 560 nm using a GloMax luminometer. Each individual experiment was repeated at least three times.

### Cell invasion assay

Cell invasion assays were performed with a BD BioCoat^™^ Matrigel^™^ Invasion Chamber (BD Biosciences, San Jose, CA, USA). The chamber contains an insert with an 8-micron pore size PET membrane coated with growth factor-reduced Matrigel. The upper chamber was seeded with 2.5x10^4^ cells suspended in serum-free RPMI 1640 medium, and the lower chamber contained RPMI 1640 medium with 5% FBS. After 24 hour incubation, non-invasive cells on the upper surface of the membrane were removed and the membrane was fixed with methanol and stained with 1x Giemsa staining solution (Sigma-Aldrich) at room temperature for 1 hour. The membrane was then photographed and the total migrating cells were counted.

### Liquid colony formation assay

To evaluate anchorage-dependent colony formation ability of NSCLC cells, 500 cells were suspended in 2 ml of RPMI 1640 medium with 5% FBS in a 6-well plate. After 2-week incubation, the cells were fixed and stained with 0.05% crystal violet (Sigma-Aldrich, Merck KGaA). Number of colonies containing more than 50 cells was counted. Each individual experiment was repeated at least three times.

### Transient transfection of NSCLC cells with siRNA

NSCLC cells were plated in 6-well plates. After an overnight incubation, the cells were transfected with 20 nM of scrambled siRNA (SIC001, Sigma-Aldrich) as a negative control or siRNA targeting ALK5 (L-003929-00-0005, Dharmacon, Lafayette, CO, USA) using Lipofectamine2000 (Thermofisher, Waltham, MA, USA) according to the manufacturer’s instructions. The cells were collected 48 hours after transfection and subjected to real-time PCR to detect the silencing efficiency.

### Statistical analysis

The experiments were performed in triplicates and the results were expressed as the mean ± standard deviation. The Student *t-*test were performed using GraphPad Prism 5 software (GraphPad, San Diego, CA, USA) to test for significant differences in RT-PCR, proliferation, cell invasion, colony formation assays. The difference was statistically significant when *P* value was lower than 0.05.

## Results

### TGFβ1 induces EMT in TGFβ1 sensitive NSCLC cell lines

**Fig 1:**
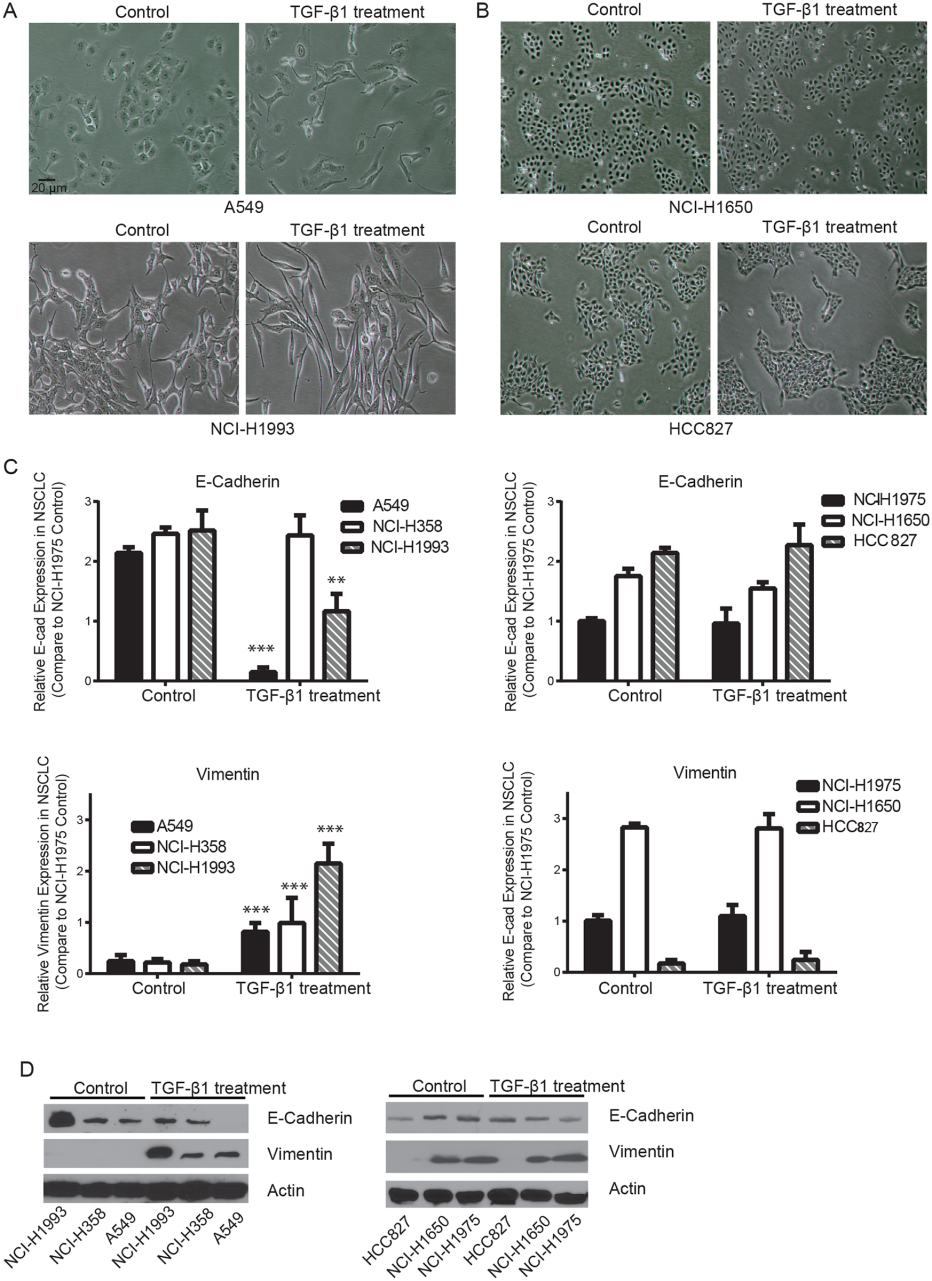
TGFβ1 induces EMT in certain non-small cell lung cancer cell lines. **(A)** A549 and NCI-H1993 cell images were taken before and after incubation with 2.5 ng/ml human recombinant TGFβ1 for two weeks. They transited from epithelial to mesenchymal like cells. Bar: 20 μm. **(B)** Morphology changes of NCI-H1650 and HCC827 cells were not noted after TGFβ1 treatment. **(C)** Real-time PCR revealed that 2.5 ng/ml TGFβ1 treatment for two weeks significantly reduced E-cadherin mRNA expression in A549 and NCI-H1993 cells (fold expression as compared to control NCI-H1975, ** *P*<0.01, *** *P*<0.001) but not in NCI-H358, NCI-H1975, NCI-H1650 and HCC827 cells. At the same time, Vimentin mRNA expression was significantly enhanced in A549, NCI-H358 and NCI-H1993 cells but not in NCI-H1975, NCI-H1650 and HCC827 cells. **(D)** Western blotting confirmed that TGFβ1 increased Vimentin and reduced E-cadherin protein expression in TGFβ1 sensitive NSCLC line.

TGFβ1 has been shown to correlate with poor prognostics in NSCLC patients [13]. In order to investigate the roles of TGFβ1 during NSCLC progression, we treated many NSCLC cell lines bearing different mutations and observed that certain NSCLC cell lines exhibited more sensitivity than the others. The most sensitive NSCLC cell lines to TGFβ1 incubation, A549, NCI-H358 and NCI-H1993, were chosen for further study. Representative data of morphology, immunoblotting, cell viability and liquid colony formation of TGFβ1 sensitive lines as well as the insensitive lines were shown. Fig 1A showed a significant morphological change before and after TGFβ1 treatment of two cell lines, A549 and NCI-H1993. Under normal cell culture conditions, A549 and NCI-H1993 cells were classical epithelial-like cells and clustered together. After 2.5 ng/ml TGFβ1 incubation for two weeks, A549 and NCI-H358 cells gradually transited to mesenchymal-like cells. They became elongated and grew individually. However, another group of NSCLC lines, such as NCI-H1650, HCC827 and NCI-H1975, were not sensitive to TGFβ1 treatment. They remained as epithelial-like cells and no significant difference on morphology was noted (Fig 1B).

To validate epithelial to mesenchymal transition in TGFβ1 sensitive NSCLC lines, we examined the expression of EMT markers before and after TGFβ1 treatment. Real-time PCR revealed that epithelial marker E-cadherin mRNA expression in A549 and NCI-H1993 were significantly reduced after 2.5 ng/ml TGFβ1 treatment for two weeks. At the same time, mesenchymal marker Vimentin mRNA expression dramatically increased (Fig 1C). Interestingly, TGFβ1 enhanced Vimentin expression in NCI-H358 cells, and did not affect the E-cadherin expression. While in TGFβ1 insensitive cells, such as NCI-H1650, HCC827 and NCI-H1975, TGFβ1 treatment showed little effects on both E-cadherin and Vimentin expression (Fig 1C). Consistently, western blotting revealed that TGFβ1 treatment enhanced Vimentin and reduced E-cadherin protein expression only in TGFβ1 sensitive NSCLC cells but not in TGFβ1 insensitive NSCLC cells (Fig 1D). These results suggest that not all NSCLC cells are responsive to TGFβ1 treatment and TGFβ1 is able to induce EMT in sensitive NSCLC cells.

### TGFβ1 reduces cell proliferation and enhances cell invasion in TGFβ1 sensitive NSCLC cell lines

**Fig 2:**
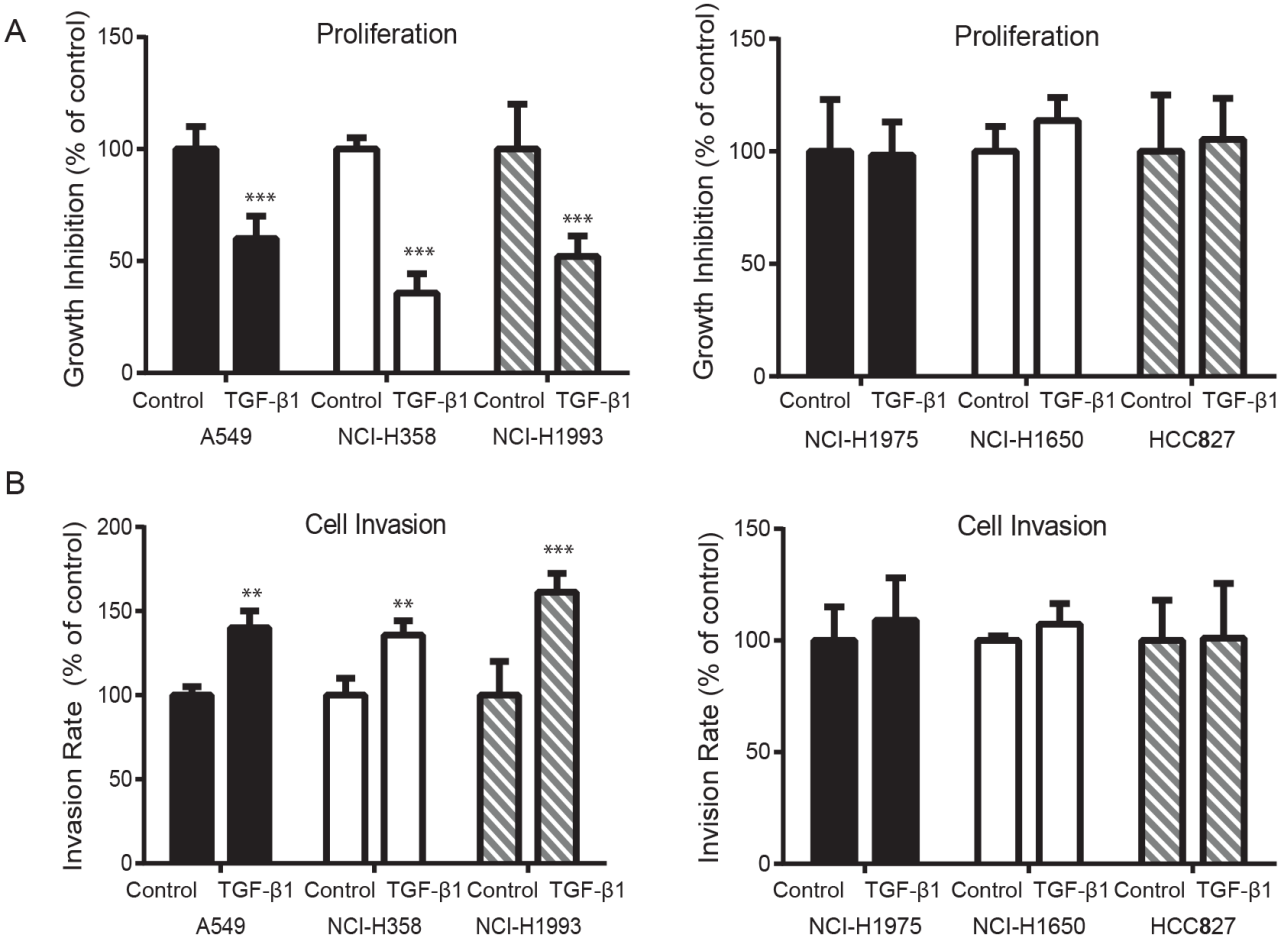
TGFβ1 reduces cell viability and increases cell invasion in TGFβ1 sensitive NSCLC cell lines. **(A)** CellTiter-Glo luminescent assays showed that 2.5 ng/ml TGFβ1 treatment for two weeks significantly reduced A549, NCI-H358 and NCI-H1993 cell viability but had no effects on NCI-H1975, NCI-H1650 and HCC827 cell growth (compared to control without treatment, *** *P*<0.001). **(B)** Cell invasion assays revealed that 2.5 ng/ml TGFβ1 treatment for two weeks significantly increased the number of A549, NCI-H358 and NCI-H1993 cells which migrated through the chambers. NCI-H1975, NCI-H1650 and HCC827 cells did not exhibit the similar properties.

To investigate whether TGFβ1 plays a role in the growth of NSCLC cells, we treated several NSCLC cell lines with 2.5 ng/ml TGFβ1 for two weeks. As shown in Fig 2A, TGFβ1 significantly reduced viable A549, NCI-H1993, or NCI-H358 cells as measured by CellTiter-Glo luminescent assays. However, in TGFβ1 insensitive NSCLC cells, NCI-H1975, NCI-H1650 or HCC827, cell viability was not affected. NSCLC cell migration and invasion are important processes during lung cancer progression. To assess the effect of TGFβ1 on NSCLC cell invasion, cell invasion assays with Matrigel invasion chambers were performed. In A549, NCI-H1993, and NCI-H358 cells, TGFβ1 dramatically increased their invasiveness, whereas the invasion of NCI-H1975, NCI-H1650 and HCC827 cells were not changed (Fig 2B). These results indicate that TGFβ1 reduces cell proliferation and increases cell invasiveness only in TGFβ1 sensitive NSCLC lines.

### TGFβ1 increases NSCLC stem-like cell population and colony formation ability in TGFβ1 sensitive NSCLC cell lines

**Fig 3:**
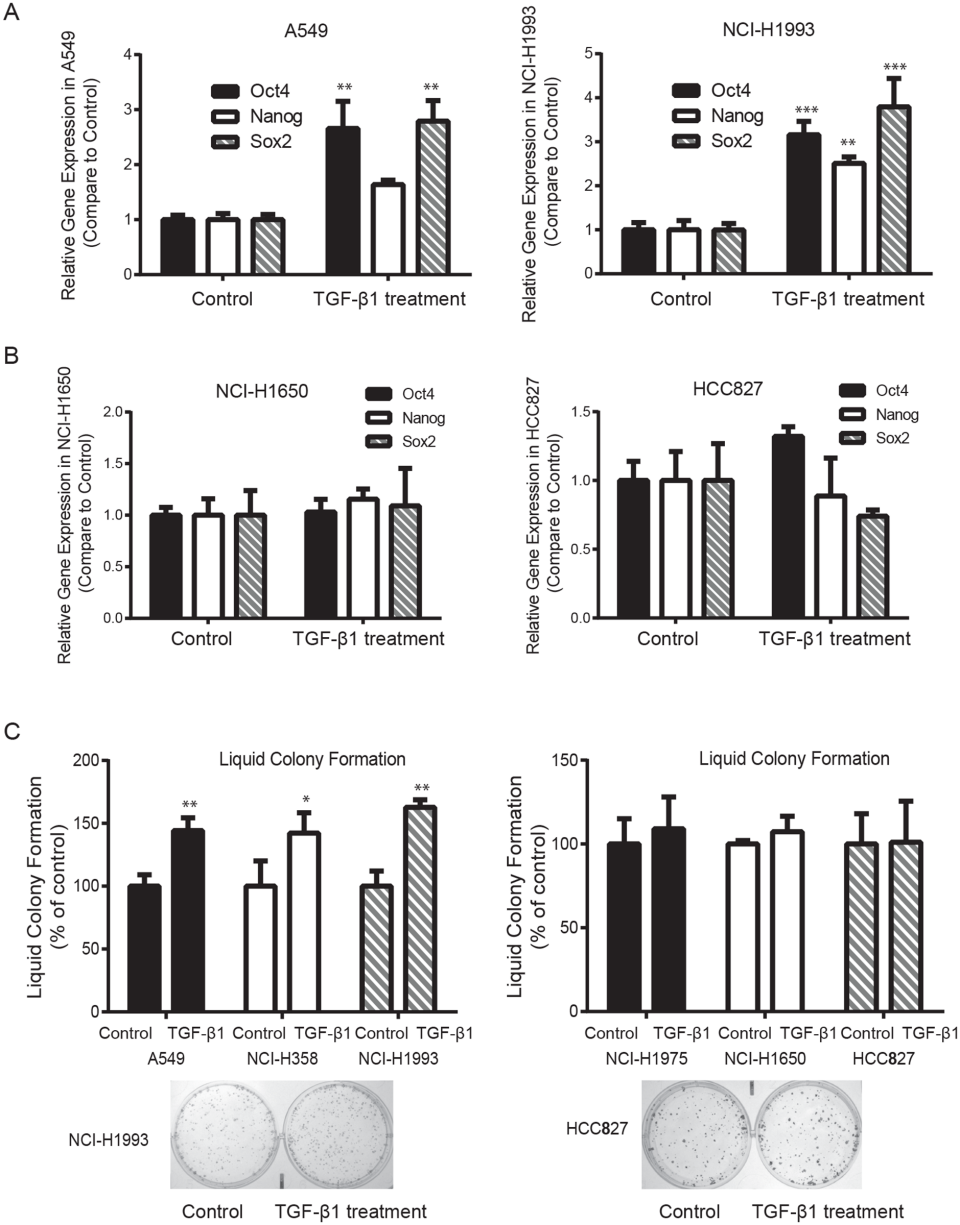
TGFβ1 increases the gene expression of NSCLC stem-like cell markers and colony formation ability in TGFβ1 sensitive NSCLC lines. **(A)** Real-time PCR showed that 2.5 ng/ml TGFβ1 treatment for two weeks significantly increased Oct4 and Sox2 mRNA expression in A549, NCI-H358 and NCI-H1993 cells compared to untreated cells (** *P*<0.01, *** *P*<0.001). **(B)** Oct4, Nanog and Sox2 expression remained same before and after TGFβ1 treatment. **(C)** 2.5 ng/ml TGFβ1 treatment for two weeks significantly enhanced anchorage-dependent colony formation ability of A549, NCI-H358 and NCI-H1993 cells but not for NCI-H1975, NCI-H1650 and HCC827 cells (* *P*<0.05, ** *P*<0.01).

Many reports demonstrated that EMT plays a role in the generation of highly invasive cells with cancer stem cell-like features, such as self-renewal and tumor formation potential, which may facilitate metastasis and tumor recurrence. To investigate whether TGFβ1 is capable of increasing NSCLC stem-like cell population, we measured mRNA expression level of cancer stem cell markers, including Oct4, Nanog, and Sox2, using real-time PCR. Fig 3A showed that 2.5 ng/ml TGFβ1 treatment for two weeks significantly increased Oct4, Nanog, and Sox2 expression in NCI-H1993 cells. In A549 cells, incubation with TGFβ1 only enhanced Oct4 and Sox2 mRNA expression but not Nanog. However, in NCI-H1975 and HCC827 cells, the expression of Oct4, Nanog, and Sox2 did not change significantly before and after TGFβ1 treatment (Fig 3B). Consistently, Fig 3C showed that 2.5 ng/ml TGFβ1 treatment results in various degree of enhancement in anchorage-dependent colony formation in A549, NCI-H1993, and NCI-H358 cells but not in NCI-H1975, NCI-H1650, and HCC827 cells. These data indicate that TGFβ1 is capable of increasing the subset of NSCLC stem-like cells and their colony forming potential in TGFβ1 sensitive cells. Further studies are required to determine whether elevated TGFβ1 level *in vivo* is involved in tumor metastasis and tumor recurrence.

### TGFβ1 increases VEGF-C expression in TGFβ1 sensitive NSCLC cell lines

**Fig 4:**
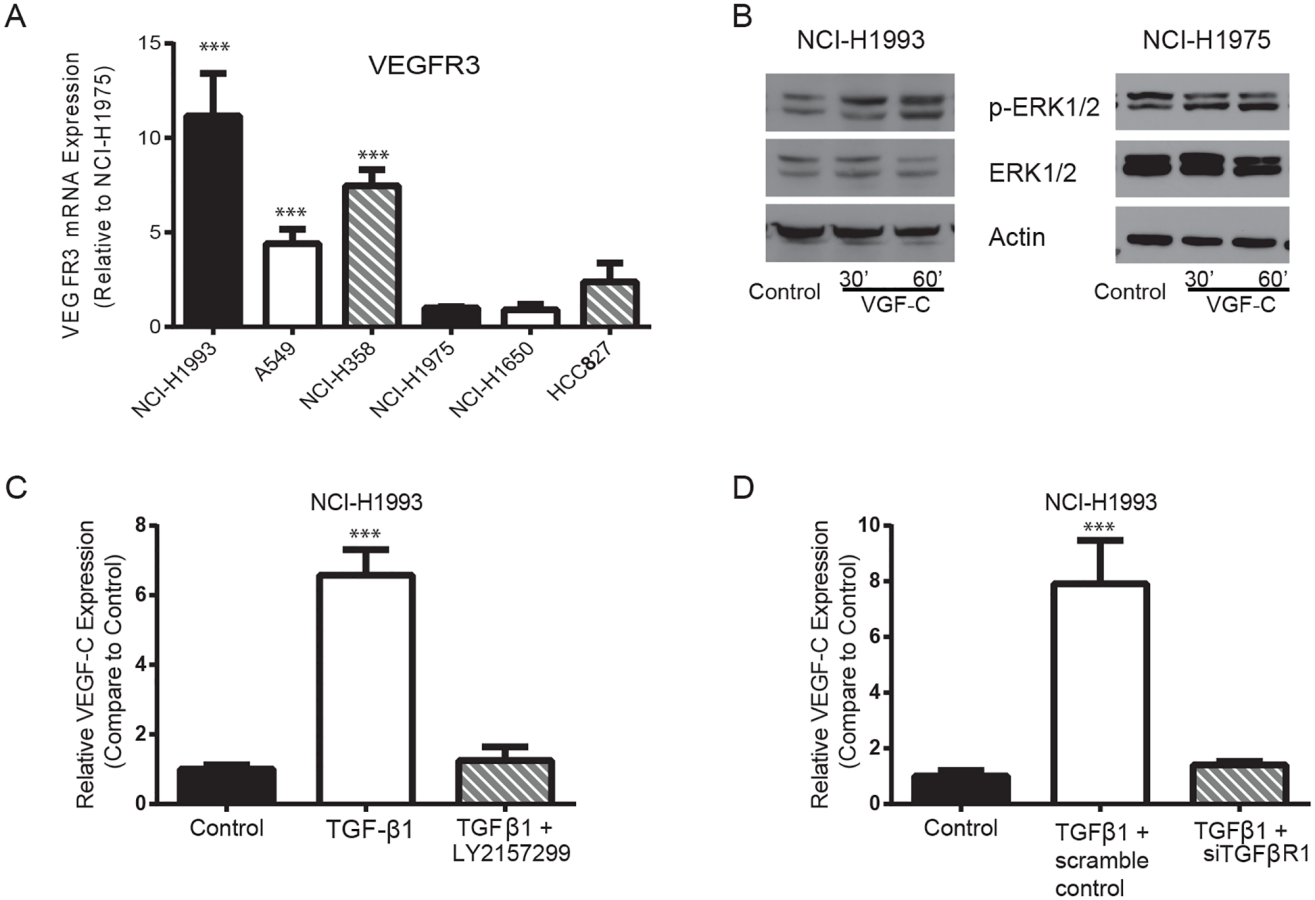
TGFβ pathway is involved in the regulation of VEGF-C expression in TGFβ1 sensitive NSCLC lines. **(A)** Real-time PCR showed that VEGFR3 mRNA expression levels were much higher in A549, NCI-H358 and NCI-H1993 cells than that in NCI-H1975, NCI-H1650 and HCC827 cells (fold expression as compared to control NCI-H1975, *** *P*<0.001). **(B)** Immunoblotting showed that human recombinant VEGF-C 10 ng/ml treatment for 30 and 60 minutes activated ERK pathway in NCI-H1993 cells but not in NCI-H1975 cells. **(C)** Real-time PCR revealed that 2.5 ng/ml TGFβ1 treatment significantly increased the VEGF-C mRNA expression in NCI-H1993 cells. The presence of 0.1 μM LY2157299 significantly reduced TGFβ1-induced VEGF-C expression. **(D)** Similarly, siRNA targeting TGFβR1 significantly decreased TGFβ1-induced VEGF-C expression compared to scramble control (*** P<0.001).

Based on the data above, next question was what the difference on gene expression profile between TGFβ1 sensitive and insensitive NSCLC cells is. Microarray analysis revealed that VEGFR3 expression levels are higher in TGFβ1 sensitive NSCLC cells relative to insensitive cells. To confirm the difference, real-time PCR validated that VEGFR3 mRNA expression in A549, NCI-H1993, and NCI-H358 was significantly more than that in NCI-H1975, NCI-H1650, and HCC827 cells (Fig 4A, compared to NCI-H1975). Furthermore, western blotting revealed that 10 ng/ml VEGF-C treatment for 30 and 60 minutes activated ERK signaling pathway in NCI-H1993 cells but not in NCI-H1975 cells (Fig 4B).

It has been shown that TGFβ1 can increase VEGF-C expression in certain cancer cells via Smad2/3 binding to the VEGF-C promoter region. This raised next question whether TGFβ1 pathway is involved in the upregulation of VEGF-C in TGFβ1 sensitive NSCLC cells. Fig 4C showed that 2.5 ng/ml TGFβ1 significantly increased VEGF-C mRNA expression in NCI-H1993 cells. Addition of 0.1 μM TGFβ receptor I kinase inhibitor LY2157299 blocked the activation of TGFβ1 pathway and drastically reduced TGFβ1-induced VEGF-C expression. To confirm the results, we employed a small interference RNA targeting TGFβR1 to knock down the expression of TGFβ receptor I. The reduction of TGFβR1 expression showed virtually the same result as the inhibitor LY2157299 (Fig 4D). These results suggest that TGFβ1 pathway is likely involved in the upregulation of VEGF-C expression in TGFβ1 sensitive NSCLC cells.

## Discussion

The current study shows that TGFβ1 induces epithelial to mesenchymal morphology transition in TGFβ1 sensitive NSCLC lines but not in insensitive lines. TGFβ1 not only increases the mesenchymal maker Vimentin expression, but also enhances NSCLC cell invasiveness. More importantly, TGFβ1 elevates both the expression level of cancer stem-like cell markers and anchorage-dependent colony formation ability in TGFβ1 sensitive lines. We also found that VEGFR3 is upregulated at the mRNA level in NSCLC lines as compared to TGFβ1 insensitive lines. TGFβ1 treatment increases VEGF-C gene expression likely through promoting Smad2/3 binding to VEGF-C promoter region. Pharmacological blocking TGFβ signaling pathway or silencing of ALK5 inhibits TGFβ1-induced VEGF-C expression in TGFβ1 sensitive NSCLC lines. These results suggest that TGFβ pathway is involved in NSCLC metastasis in TGFβ1 sensitive lines.

Non-small cell lung cancer is a complex and heterogeneous disease characterized by a unique molecular profile of each patient. Originally we hypothesized that TGFβ1 would induce EMT in most epithelial-like NSCLC lines. Surprisingly, only a subset of epithelial NSCLC lines was responsive to TGFβ1 treatment. There are several possible reasons: (1) different NSCLC lines were generated from various stages of lung cancer patients and the responsiveness of TGFβ signaling pathway were different during the cancer progression; (2) the combination of different key mutations in each cell line leads to various sensitivity to TGFβ1; (3) TGFβ1 responsiveness of NSCLC lines may be affected by their interaction with other players in the microenvironment. Further *in vivo* studies are important to determine the mechanisms. We noticed that NCI-H1975, NCI-H1650, and HCC827 lines have EGFR mutations. Further testing on other EGFR mutated lines will be critical to setup the relationship between EGFR mutation and TGFβ1 insensitivity and to understand the mechanism of TGFβ1 sensitivity in NSCLC.

After identify the group of TGFβ1 sensitive NSCLC lines, we performed microarray analysis and found that VEGFR3 was upregulated compared to insensitive lines. We and other labs have reported that plasma VEGF-C level is positively correlated with poor prognosis of NSCLC patients [14–18]. Regan et al. found that VEGF-C gene expression in lymphangiogenic NSCLC lines is 50 folder higher that in non-lymphangiogenic lines [19]. In this study we showed that TGFβ1 could increase VEGF-C gene expression in TGFβ1 sensitive lines. Blocking TGFβ signaling pathway activation by pharmacological inhibitors or gene silencing successfully decreased VEGF-C expression. VEGF-C and VEGF-D are known as important growth factors involved in lymphangiogenesis [20]. The upregulation of VEGF-C in tumor cells promotes sprouting of lymphatic vessels, expansion of collecting lymph vessels, and lymphatic metastasis of tumors [21–24]. Liu et al. showed that in cervical squamous carcinoma cells sine oculis homeobox homolog 1 (SIX1) promotes tumor lymphangiogenesis via coordinating with TGFβ to increase VEGF-C expression [25]. These results suggest that it is likely TGFβ1 promotes tumor metastasis partially through VEGF-C pathway.

Cancer stem cell hypothesis asserts that only a small subset of cells within a tumor or cultured cell lines is capable of self-renewal and tumor initiation. EMT is often activated during cancer invasion and metastasis. Especially reported in breast cancer cells, EMT is a critical process for cancer cells to acquire stem-like properties in order to spread the metastases [26, 27]. Pirozzi et al. reported that EMT induced by TGFβ1 increased the expression of Oct4, Nanog, Sox2, c-kit and CD133 in A549 and LC-31 cells [28]. Nobiletin and Epicatechin-3-gallate can inhibit epithelial-mesenchymal transition of human non-small cell lung cancer cells by antagonizing the TGF-beta1/Smad3 signaling pathway [29, 30]. Similarly, we found that TGFβ1 could promote the gene expression of cancer stem-like cell markers and increase cell invasiveness in TGFβ1 sensitive NSCLC lines, strongly indicating that TGFβ1 is involved in the generation of cancer stem-like cell subpopulation, which is likely associated with tumor metastasis and recurrence in NSCLC patients.

How TGFβ1 contributes to NSCLC metastasis and cancer stem-like cell generation is probably complex. In the future, identifying the molecular profile of TGFβ1 sensitive NSCLC lines and exploring the effects of TGFβ1 on lymphangiogenesis *in vivo* will lead to a better understanding of the role of TGFβ1 in lung cancer progress and eventually to TGFβ pathway inhibitors that could be used for clinical treatment.
